# Positive Selection on Loci Associated with Drug and Alcohol Dependence

**DOI:** 10.1371/journal.pone.0134393

**Published:** 2015-08-13

**Authors:** Brooke Sadler, Gabe Haller, Howard Edenberg, Jay Tischfield, Andy Brooks, John Kramer, Marc Schuckit, John Nurnberger, Alison Goate

**Affiliations:** 1 Department of Psychiatry, Washington University, St. Louis, MO, United States of America; 2 Department of Orthopedic Surgery, Washington University, St. Louis, MO, United States of America; 3 Department of Molecular Biology, Indiana University, Indianapolis, IN, United States of America; 4 Department of Genetics, Rutgers University, Piscataway, NJ, United States of America; 5 Department of Psychiatry, University of Iowa, Iowa City, IA, United States of America; 6 Department of Psychiatry, University of San Diego, La Jolla, CA, United States of America; 7 Department of Psychiatry, Indiana University, Indianapolis, IN, United States of America; 8 Department of Neuroscience, Mount Sinai, New York City, NY, United States of America; Harvard Medical School, UNITED STATES

## Abstract

Much of the evolution of human behavior remains a mystery, including how certain disadvantageous behaviors are so prevalent. Nicotine addiction is one such phenotype. Several loci have been implicated in nicotine related phenotypes including the nicotinic receptor gene clusters (*CHRN*s) on chromosomes 8 and 15. Here we use 1000 Genomes sequence data from 3 populations (Africans, Asians and Europeans) to examine whether natural selection has occurred at these loci. We used Tajima’s D and the integrated haplotype score (iHS) to test for evidence of natural selection. Our results provide evidence for strong selection in the nicotinic receptor gene cluster on chromosome 8, previously found to be significantly associated with both nicotine and cocaine dependence, as well as evidence selection acting on the region containing the *CHRNA5* nicotinic receptor gene on chromosome 15, that is genome wide significant for risk for nicotine dependence. To examine the possibility that this selection is related to memory and learning, we utilized genetic data from the Collaborative Studies on the Genetics of Alcoholism (COGA) to test variants within these regions with three tests of memory and learning, the Wechsler Adult Intelligence Scale (WAIS) Block Design, WAIS Digit Symbol and WAIS Information tests. Of the 17 SNPs genotyped in COGA in this region, we find one significantly associated with WAIS digit symbol test results. This test captures aspects of reaction time and memory, suggesting that a phenotype relating to memory and learning may have been the driving force behind selection at these loci. This study could begin to explain why these seemingly deleterious SNPs are present at their current frequencies.

## Introduction

Consequences of nicotine dependence are the leading cause of preventable death in the USA and it has been noted that some populations experience higher levels of addiction than others [1] but the reason for this is not understood. Multiple studies have demonstrated a genetic component to nicotine addiction [2–4], but little is known about the role of natural selection in shaping the genetic components of nicotine addiction. Such knowledge could help us understand the genetic and behavioral nature of addiction and ultimately facilitate the design and delivery of appropriate interventions to reduce nicotine addiction.

It has been estimated that approximately 10% of the genome has been affected by linkage due to recent selective sweeps [5]. However, it is challenging to make direct and clear inference on the phenotypic that is the target of natural selection. This is particularly true when the phenotype being examined has no obvious evolutionary consequences, and has a disease or trait-related association with no clear reproductive consequence today, but where patterns of genetic variation are consistent with a positive selective sweep in recent human history. In this case, additional mechanisms and/or alternative explanations must be sought for the existence of selection on the gene of interest. An example of such a situation occurs in the gene encoding hemoglobin. In homozygous form, the ‘sickle cell’ allele, HbS, drives the formation of malformed red cells, which aggregate, blocking blood flow to numerous organs including the brain. This results in organ damage and strokes, severely shortening the lifespan of the individual. Nonetheless, the HbS allele is maintained in the gene pool in regions where malaria is endemic because in heterozygous form it provides protection against malaria (for a review see [6]. The case of nicotine addiction represents a similar conundrum. Several genetic variants that modify susceptibility to or protection from nicotine dependence have been identified by genome-wide association studies (GWAS) [2,4,7]. Perhaps not surprisingly, the loci identified in these studies mainly include genes encoding neuronal nicotinic cholinergic receptors (*CHRNs*).

Neuronal cholinergic nicotinic receptors *(CHRNs)* are a heterogeneous class of cation (positively charged) channels expressed in the central and peripheral nervous system. There are 11 neuronal *CHRN* genes, each of which encodes a receptor subunit. The neuronally expressed nicotinic receptors consist of combinations of alpha and beta subunits, encoded in humans by 8 alpha (α2-α 7, α9-α10) and 3 beta (β2-β4) genes [8]. These subunits form homo- or hetero-pentameric subtypes, which are present in various regions throughout the nervous system. To form a receptor, five subunits must be combined within the cell and the specific combination of these subunits defines the receptor subtype.

In the body, the opening of these channels is controlled by the endogenous ligand, acetylcholine, a chemical produced by neurons to activate other nearby neurons. Nicotine, the major psychoactive chemical present in tobacco smoke is a chemical present in the environment that can also stimulate the opening of these nicotinic acetylcholine receptor ion channels [9]. A number of GWAS studies have been performed that demonstrate an association between the nicotinic receptors and smoking. The strongest association between nicotinic receptors and nicotine addiction is a non-synonymous change (rs16969968, D398N) in the gene encoding the α5 subunit of the nicotinic receptor *(CHRNA5)* on chromosome 15 [7,10–14]. When cells are made to express nicotinic receptors containing the minor allele form of this SNP (398N), agonists induce less channel opening and cell activation than in cells that express receptors containing the major allele (398D) [11]. Thus, the minor allele at this SNP results in a significant functional change in the behavior of this ion channel, causing more nicotine to be needed in individuals with the minor allele to produce the same effect. Additionally, SNPs within the chromosome 15 and chromosome 8 regions have been associated with alocohol and cocaine dependence in addition to their associations with smoking related traits [15–17]. Although the underlying functional mechanism underlying the associations at the nicotinic receptors on chromosome 8 is not known, there is nevertheless a genome-wide significant signal with the LD bin [18].

Here we test the hypothesis that natural selection has acted on these genes. But if the null hypothesis of neutral evolution or demographic processes is rejected, why would nature seemingly select for this trait, especially given the fact that it is believed that nicotine has not been a part of our evolutionary history long enough, and in large enough quantities, for its effects to be visible in our genomes? One hypothesis is that selection acted on another phenotype and the effect on nicotine addiction was secondary and incidental, a genetic phenomenon termed hitchhiking.

Nicotine is known to have an enhancing effect on cognitive performance. It enhances the reorientation of attention in visuospatial tasks [19] and alters the neuronal activity responsible for increased attention and arousal [20]. Furthermore, several studies have found associations between SNPs in multiple nicotinic receptor genes and cognitive performance [21,22]. However, effects on cognitive performance at SNPs related to nicotine dependence seem peculiar given the presumed acceleration in cognitive development over recent human history. Therefore, these results suggest that if selection is shaping the genetic landscape of these genes, it may be through their effect on cognitive function in the absence of drug use. Evidence from nicotinic receptor knockout mice also supports a role for nicotinic receptors in memory and learning, as well as anxiety levels. *CHRNA7* knockout mice have impaired reaction times [23] and decreased procedural learning [24], while, *CHRNA6* knockout mice show that this receptor plays a role in nicotinic modulation of dopaminergic transmission, an important component of learning and memory [25]. Based on these observations, we hypothesize that at least some of the nicotinic receptors may have been targets of recent selection and that this selection is related to the role of nicotinic receptors in memory and learning.

To test this hypothesis, we use two summaries of genetic variation that have different statistical power to make inferences, depending on the model of selection and associated population demography. Two different methods were used for detecting natural selection at two loci relevant to nicotine dependence, specifically the *CHRNA5-A3-B4* region on chromosome 15q25 and the *CHRNB3-A6* region on chromosome 8p11. We provide strong evidence for selection in the *CHRNB3-A6* region and moderate evidence for selection in the *CHRNA5-A3-B4* region. However, there is only a modest correlation between nicotine dependence and score on the Wechsler Adult Intelligence Scale (WAIS) Digit Symbol test in our dataset. Overall, we suggest that one possible explanation for these results is that SNPs in these regions associated with risk of nicotine dependence are associated with natural selection acting at these loci to improve human memory and learning.

## Results

### Tajima’s D Test

**Fig 1:**
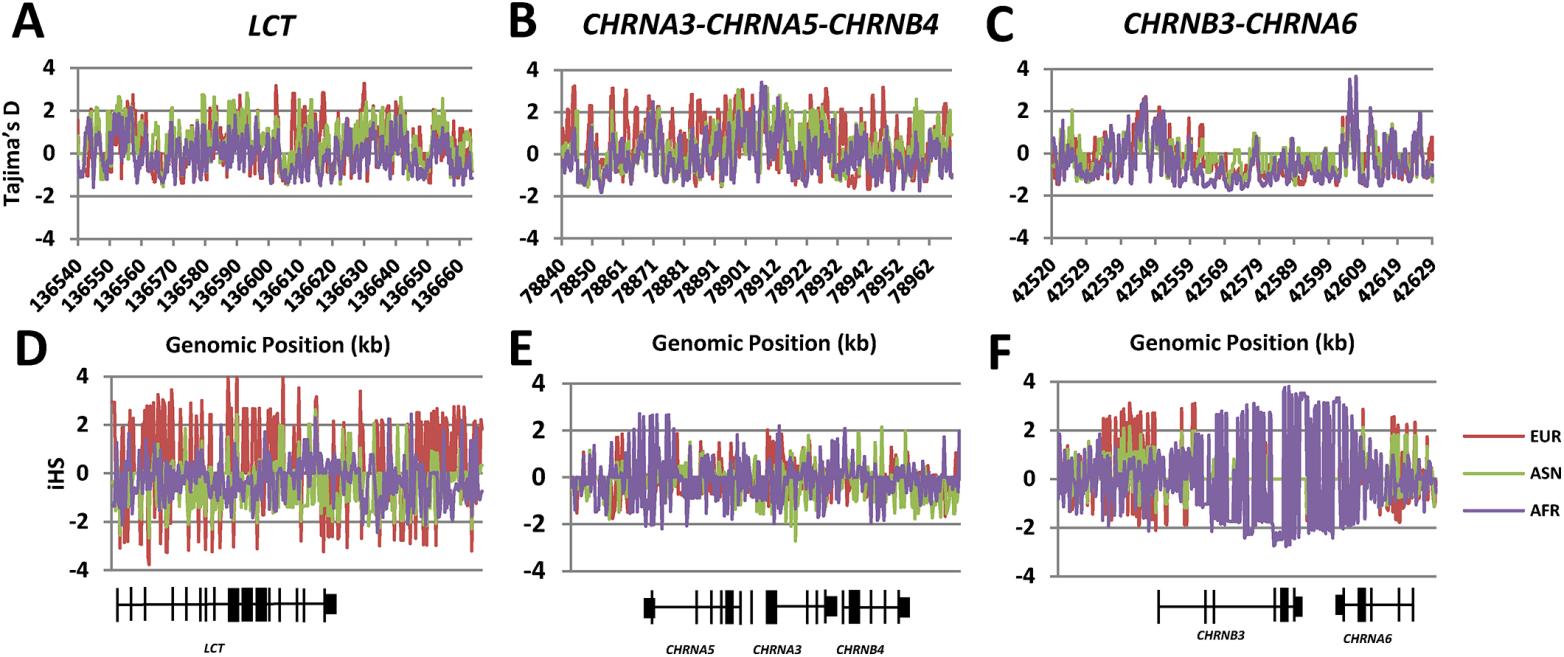
Tajima’s D and iHS Results. A-CTajima’s D andD-F) iHS for the *LCT* region on chromosome 2, the *CHRNA3-B4-A5* gene cluster on chromosome 15 and the *CHRNB3-A6 gene* cluster on chromosome 8 across individuals of European (red), African (Purple) or Asian (green) ancestry. Lines are the 95% confidence intervals as calculated by permutation for iHS and Tajima’s D.

We calculated Tajima’s D over the regions of the nicotinic receptors on chromosomes 8 and 15, as well as lactase on chromosome 2 as the positive control. As the program utilized does not incorporate ancestral information, all results are based on the folded frequency spectrum, i.e. the distribution of polymorphic sites according to the number of chromosomes that carry a given minor allele rather than the number of chromosomes that carry the derived, non-ancestral allele. [26]Fig 1 shows regional plots of the Tajima’s D values for the *LCT*, *CHRNA5-A3-B4* nicotinic receptor gene region and the *CHRNA6-B3* gene region. Within each of these regions, we calculated sliding windows of 10 kb with an increment of 1 kb. We then compared the number of windows within each region with Tajima’s D values above 2 or below -2 (this represents the 95% confidence interval of values in our data) to permutations of 10,000 randomly selected regions of the same size across the genome.

We utilized the *LCT* gene region as a positive control. *LCT* encodes the protein lactase and mutations in the region give rise to lactase persistence. The lactase persistence phenotype is due to changes in a regulatory region that enhances the expression of *LCT* located in intron 13 of the neighboring gene, *MCM6* [27]. Fig 1A shows the distribution of Tajima’s D values in the sliding windows in the *LCT* region in AFR, ASN and EUR. As expected, both the Asian and European populations show many windows above 2 but very few exist in the African population. All p-values are derived using permutations of 10,000 regions of the same size from across the genome. For Europeans and Asians, the proportion of windows with extreme values was 8.6% and 6.5%, respectively (Table 1). This is highly significant in EUR, when compared to the negative control regions, and modestly significant in ASN (<p = 2x10^-7^ and p = 0.01 for EUR and ASN, respectively) and appears inconsistent with the null hypothesis of neutral evolution and demography at the *LCT* locus especially in the EUR populations. In contrast, the histogram for AFR shows few windows with extreme Tajima’s D values. Indeed, the proportion of windows with extreme values was <1% and was not significantly different from the negative controls, therefore we are unable to reject the null hypothesis of neutral evolution and demography at this locus in AFR.

**Table 1:** Summary of Extreme Tajima’s D and iHS Values in Each Region in Comparison with Negative Controls.

Gene(s)	Pop	# windows|TajD|>2	Proportion	p-value	# windows|iHS|>2	Proportion	p-value
*CHRNA5-A3-B4*	EUR	173/1273	13.60%	***<2 x 10*** ^***−16***^	1/181	<1%	NS
	ASN	91/1273	7.10%	***4 x 10*** ^***−4***^	3/224	1.3%	NS
	AFR	30/1294	2.30%	NS	16/266	5.6%	NS
*CHRNB3-A6*	EUR	13/1066	1.22%	NS	26/110	23.6%	***4 x 10*** ^***−4***^
	ASN	1/973	<1%	NS	3/116	2.59%	NS
	AFR	23/1097	2.10%	NS	93/357	26.1%	***3 x 10*** ^***−4***^
*LCT/MCM6*	EUR	98/1136	8.63%	***2 x 10*** ^***−7***^	111/138	80.4%	***< 1 x10*** ^***-6***^
	ASN	76/1161	6.55%	***0*.*01***	20/219	9.1%	NS
	AFR	5/1240	<1%	NS	9/278	3.2%	NS

Proportion of extreme Tajima’s D and his values in selected regions. P-values are produced by permutation of 10,000 randomly selected regions of the same size across the genome.

We next measured Tajima’s D in the *CHRNA5-A3-B4* gene cluster on chromosome 15. In this region, we analyzed a 120 kb region that includes the nicotinic receptor gene cluster as well as ~18 kb upstream of *CHRNA5*. This was done to ensure that we included the large region upstream of *CHRNA5* that has been associated with regulation of the level of mRNA expression for *CHRNA5* [28]. Fig 1B shows the distribution of Tajima’s D values in the sliding windows in the *CHRNA5-A3-B4* region in AFR, ASN and EUR. Overall, we observed increased numbers of sliding windows with extreme Tajima’s D values in each of the three populations, which is not consistent with a neutral evolution and demography in this region (Table 1). Of note, several SNPs previously shown to be associated with nicotine, alcohol and/or cocaine dependence [7,10–14] are within regions with high Tajima’s D values. Additionally, within the EUR population, these regions also harbor SNPs in an LD bin tagged by rs5887655 previously shown to be significantly associated with *CHRNA5* mRNA expression in the brain [28]. Table 2 summarizes the Tajima’s D values for windows containing associated SNPs in each of these two LD bins.

**Table 2:** Tajima’s D and iHS values for *CHRNA5-A3-B4* region.

SNP	Function	EUR Freq	Tajima’s D EUR	iHS EUR	ASN Freq	Tajima’s D ASN	iHS ASN	AFR Freq	Tajima’s D AFR	iHS AFR
rs72740955	intergenic	0.37	(1.53, 2.07)	-0.74	0.03	(0.72, 0.97)	-1.25	0.09	(0.88, 1.00)	0.44
rs2036527	intergenic	0.37	(0.11, 0.11)	-0.5	0.03	(-0.57, -0.54)	-0.85	0.16	(-1.15, 0.20)	1.48
rs55853698	CHRNA5 5'UTR	0.37	(0.56, 1.93)	-0.65	0.04	(-0.02, 1.00)	-0.31	0.06	(0.24, 0.56)	1.05
rs17486195	CHRNA5 intronic	0.36	(1.59, 1.64)	-0.62	0.03	(-0.17, 0.14)	-0.82	0.11	(-0.10, -0.07)	0.48
rs17486278	CHRNA5 intronic	0.36	(0.39, 0.70)	-0.62	0.31	(0.04, 0.60)	-0.45	0.28	(0.42, 0.47)	0.72
rs72740964	CHRNA5 intronic	0.36	(0.78, 1.63)	-0.73	0.03	(0.21, 0.98)	-0.57	0.04	(0.08, 0.56)	0.14
rs951266	CHRNA5 intronic	0.36	(-0.22, 0.22)	-0.65	0.03	(-0.84, -0.37)	-0.5	0.08	(-0.89, -0.74)	0.91
**rs16969968**	CHRNA5 missense	0.36	**(2.00, 2.33)**	-0.71	0.03	(0.43, 1.01)	-0.44	0.02	(0.29, 0.62)	-0.01
rs1051730	CHRNA3 synonymous	0.36	(1.40, 1.90)	-0.64	0.03	(0.41, 0.78)	-0.67	0.09	(-0.29, -0.23)	0.69
**rs1317286**	CHRNA3 intronic	0.36	(0.32, 1.27)	-0.8	0.09	**(2.00, 2.56)**	0.78	0.24	(0.73, 1.36)	-0.43
**rs12914385**	CHRNA3 intronic	0.4	**(2.35, 2.55)**	-0.39	0.32	(1.85, 2.01)	0.36	0.2	(1.13, 1.35)	0.39
rs114205691	CHRNA3 intronic	0.64	(0.80, 1.65)	1.53	0.68	(0.00, 0.81)	0.15	0.8	(1.75, 1.97)	0.23
rs8040868	CHRNA3 synonymous	0.59	(0.87, 1.37)	1.54	0.62	(1.63, 2.17)	0.25	0.63	(-0.27, 0.13)	0.67
rs55958997	intergenic	0.39	(1.03, 1.66)	-1.08	0.06	(0.74, 1.23)	0.23	0.3	(-0.41, -0.38)	-0.45
rs72743158	CHRNB4 intronic	0.38	(0.26, 1.27)	-0.79	0.02	(0.79, 1.34)	-0.83	0.03	(0.20, 0.20)	1.11
rs55988292	CHRNB4 intronic	0.39	(-1.52, -0.24)	-0.26	0.02	(-0.35, 028)	-0.94	0.14	(-0.93, -0.61)	-0.7
**rs4275821**	intergenic	0.33	**(2.02, 2.32)**	-0.39	0.14	(0.61, 088)	-0.02	0.18	(0.74, 0.99)	-0.13
**rs588765**	CHRNA5 intronic	0.4	**(2.18, 2.22)**	-0.32	0.15	(0.00–0.26)	-0.2	0.25	(-0.30, -0.30)	0.44
**rs6495306**	CHRNA5 intronic	0.4	**(2.07, 2.32)**	-0.32	0.15	(-0.42, 0.06)	-0.2	0.26	(-0.16, 0.31)	0.41
rs495090	CHRNA5 intronic	0.35	(1.47, 2.06)	-0.66	0.2	(1.17, 1.37)	0.48	0.4	(0.77, 1.23)	0.06
rs680244	CHRNA5 intronic	0.4	(-0.77, 0.83)	-0.27	0.21	(-0.60, 0.24)	0.62	0.4	(-0.63, -0.06)	-0.11
**rs621849**	CHRNA5 intronic	0.41	(0.36, 1.35)	-0.27	0.21	**(2.07, 2.08)**	0.57	0.4	(0.47, 1.13)	-0.21
rs11637635	CHRNA5 intronic	0.65	(1.61, 2.18)	1.51	0.86	(-0.28, -0.13)	0.99	0.78	(-0.60, -0.35)	0.35
rs481134	CHRNA5 intronic	0.6	(0.14, 0.77)	1.1	0.85	(-0.48, 0.19)	1.06	0.75	(-0.87, -0.61)	0.19
rs555018	CHRNA5 intronic	0.4	(-0.84, 0.78)	-0.22	0.15	(-0.39, 0.04)	0.01	0.26	(-0.77, -0.23)	0.97
rs647041	CHRNA5 intronic	0.4	(-1.06, -0.14)	-0.24	0.18	(-1.05, 0.03)	0.24	0.24	(-1.08, -0.60)	0.77
**rs615470**	CHRNA5 3' UTR	0.65	**(2.54, 2.57)**	1.46	0.83	(-1.20, -0.75)	0.66	0.67	(-1.09, -0.60)	-0.08
rs6495307	CHRNA3 intronic	0.6	(-1.17, 0.14)	0.96	0.82	(0.55, 0.84)	0.71	0.66	(-0.83, -0.41)	-0.07
rs62010327	CHRNA3 intronic	0.35	(0.15, 1.60)	-0.74	0.14	(0.42, 0.85)	-0.78	0.08	(0.65, 0.97)	-0.99
rs12901300	CHRNA3 intronic	0.4	(-1.37, -0.26)	-0.3	0.19	(-0.71, 0.11)	0.26	0.34	(-0.47, 0.32)	0.91
rs3743077	CHRNA3 intronic	0.4	(1.86, 2.62)	-0.27	0.18	(-0.25, 0.14)	0.34	0.11	(-0.96, -0.77)	0.33
rs62010328	CHRNA3 intronic	0.34	(1.86, 1.90)	-0.8	0.14	(-0.54, -0.25)	-0.69	0.07	(-1.17, -0.96)	-0.54
**rs2869546**	CHRNA3 intronic	0.36	**(2.08, 2.40)**	-0.18	0.18	**(2.31, 2.63)**	-0.09	0.29	**(3.17, 3.41)**	0.66
rs4366683	CHRNA3 intronic	0.54	(-0.72, 0.13)	0.44	0.45	(1.50, 2.00)	-0.96	0.5	(0.70, 1.54)	0.02
rs58643100	CHRNA3 intronic	0.46	(-0.72, 0.13)	0.63	0.26	(1.50, 2.00)	0.84	0.41	(0.70, 1.54)	0.24

All SNPs have r^2^ value of 0.8 or greater with rs16969968 or rs588765. Significant values and SNP names are bolded.

For the *CHRNB3-A6* region, we examined a rather larger segment of the genome upstream of the gene cluster. This was done in order to include several upstream SNPs that have previously been shown to exhibit associations with nicotine dependence or cocaine dependence [17]. Fig 1C shows a regional plot of Tajima’s D values across the sliding windows in the *CHRNB3-A6* region. In AFR, there were several windows with extreme Tajima’s D values upstream of *CHRNB3* and in the intergenic region between *CHRNB3* and *CHRNA6*. Among the three populations tested, none showed a significant increase in the number of windows with extreme Tajima’s D values when compared to the permutations. The concentration of windows with extreme Tajima’s D values upstream of *CHRNB3* is noteworthy in the context of risk for nicotine addiction. Of note, a recent genome-wide association study found that a SNP in this region, rs1451240, was associated with reduced risk for nicotine dependence, measured using the Fagerstrom Test for Nicotine Dependence (FTND) [18]. The LD bin tagged by rs1451240 spans ~66 kb and several other alleles of SNPs in this bin have been associated with reduced risk for nicotine dependence, although no others are significant at the genome-wide level [18]. The data in Table 3 show that in EUR and AFR, four adjacent SNPs from this LD bin, including rs1451240, were present in sliding windows with extreme Tajima’s D values in AFR. These data suggest that these nicotine dependence-associated SNPs may be undergoing balancing selection or positive selection in these two populations.

**Table 3:** Tajima’s D and iHS values for *CHRNB3-CHRNA6* region.

SNP	Function	EUR Freq	Tajima's D EUR	iHS EUR	ASN Freq	Tajima's D ASN	iHS ASN	AFR Freq	Tajima's D AFR	iHSAFR
rs1979140	intergenic	0.77	(-0.75, -0.34)	-1.44	0.82	(-1.21, -1.15)	-0.47	0.28	(-1.26, -0.99)	-0.15
rs7816726	intergenic	0.77	(0.33, 1.00)	-1.44	0.81	(-0.02, 0.70)	-0.49	0.28	(0.08, 0.17)	-0.56
**rs10958726**	intergenic	0.23	(0.1, 0.68)	**2.46**	0.18	(-0.85, -0.15)	1.06	0.63	(-0.70, 0.18)	0.65
**rs7842601**	intergenic	0.23	(0.17, 0.62)	**2.58**	0.18	(-0.33, -0.17)	1.06	0.63	(0.63, 1.33)	0.84
**rs13273442**	intergenic	0.23	(-0.39, 0.69)	**2.32**	0.18	(-0.60, -0.45)	1.02	0.63	(-0.75, 0.04)	-0.21
rs9792277	intergenic	0.77	(1.56, 1.80)	-1.45	0.81	(1.06, 1.17)	-0.54	0.33	(1.38, 2.32)	0.77
**rs1451239**	intergenic	0.77	**(2.41, 2.58)**	-1.45	0.82	(1.34, 1.40)	-0.46	0.34	**(2.18, 2.51)**	0.89
**rs1451240**	intergenic	0.23	**(2.41, 2.54)**	**2.48**	0.18	(1.33, 1.41)	1.11	0.67	**(2.18, 2.46)**	0.09
**rs1901281**	intergenic	0.77	**(2.37, 2.68)**	-1.56	0.81	(1.26, 1.33)	-0.54	0.28	**(2.18, 2.19)**	0.37
**rs4736835**	intergenic	0.23	**(2.48, 2.68)**	**2.6**	0.18	(1.17, 1.26)	1.11	0.66	(1.47, 2.63)	-0.08
rs1955185	intergenic	0.77	(1.49, 1.67)	-1.58	0.82	(0.64, 0.69)	-0.52	0.28	(1.08, 1.59)	0.54
**rs13277254**	intergenic	0.23	(0.89, 1.27)	**2.69**	0.18	(0.64, 0.97)	1.32	0.63	(1.11, 1.29)	-0.6
rs13277524	intergenic	0.77	(0.64, 0.89)	-1.63	0.82	(0.24, 0.97)	-0.65	0.28	(0.43, 1.29)	0.61
rs6474412	intergenic	0.77	(1.09, 1.43)	-1.63	0.82	(0.53, 0.66)	-0.65	0.34	(1.01, 1.49)	1.25
rs6474413	intergenic	0.77	(1.86, 2.19)	-1.66	0.82	(0.13, 0.22)	-0.65	0.28	(1.15, 1.33)	0.62
**rs7004381**	intergenic	0.23	(0.68, 1.86)	**2.72**	0.18	(-0.15, 0.22)	1.32	0.63	(0.29, 1.15)	-0.72
rs6985052	intergenic	0.77	(-0.37, 0.68)	-1.66	0.82	(-0.85, -0.16)	-0.65	0.29	(-1.04, -0.29)	0.71
**rs4950**	CHRNB3 5'UTR	0.23	(-0.39, 0.12)	**2.71**	0.18	(-0.44, -0.16)	1.31	0.8	(0.38, 1.55)	0.25
rs9643891	CHRNB3 intronic	0.77	(-0.34, 0.13)	-1.85	0.82	(0.60, 0.72)	-1.01	0.15	(-0.65, -0.16)	-0.15
rs9643853	CHRNB3 intronic	0.77	(-1.04, -0.34)	-1.89	0.82	(-0.60, 0.60)	-1.01	0.15	(-1.55, -0.65)	-0.1
**rs13280604**	CHRNB3 intronic	0.23	(0.47, 1.68)	**2.9**	0.18	(0.07, 0.50)	1.94	0.8	(0.04, 0.83)	0.12
rs6997909	CHRNB3 intronic	0.77	(0.70, 0.70)	-1.88	0.82	(-0.25, 0.11)	-1.19	0.15	(-1.00, -1.00)	0
rs6474414	CHRNB3 intronic	0.77	(0.40, 0.40)	-1.88	0.82	(-0.60, -0.21)	-1.19	0.15	(-0.76, -0.12)	0
**rs6474415**	CHRNB3 intronic	0.23	(1.26, 1.35)	**3.07**	0.18	(-0.12, 0.22)	1.97	0.85	(-0.85, -0.52)	0.84
rs4236926	CHRNB3 intronic	0.77	(-0.04, -0.03)	-1.71	0.81	(-0.14, -0.11)	-1.41	0.15	(-0.54, -0.54)	0.14
rs16891561	CHRNB3 intronic	0.77	(-1.28, -0.90)	-1.67	0.81	(-0.89, 0.72)	-1.26	0.15	(-1.08, -0.48)	0.2
**rs55828312**	CHRNB3 intronic	0.24	(-(0.89, -0.74)	**2.86**	0.19	(-1.13, -0.31)	2.13	0.83	(-0.85, 0.67)	1.12

All SNPs have r^2^ value of 0.8 or greater with rs1451240. Significant values and SNP names are bolded.

### Integrated Haplotype Score (iHS) Analyses

Fig 1 shows regional plots of iHS scores for the *LCT*, *CHRNA5-A3-B4* nicotinic receptor gene region and *CHRNA6-B3* nicotinic receptor gene region. Within each of these regions, we calculated the iHS score for all SNPs for which we could unambiguously determine the chimp ancestral allele. We then compared the number of SNPs within each region with iHS values above 2 or below -2, to the 10,000 equally sized permutated regions that were chosen at random.

We again used the *LCT/MCM6* region as a positive control. Fig 1D shows the regional plots of iHS values in the *LCT/MCM6* region for EUR, ASN and AFR populations. As expected, the AFR population shows few extreme values (only 3.2%) and this does not differ significantly from the negative control. In EUR and ASN, the overall average proportion of extreme values for this region is 80.4% and 9.1%, respectively (Table 1). The clustering of extreme iHS values in the genic areas of this region is consistent with what is known about large-scale positive selection at this locus in the EUR population and to a lesser extent in the ASN population [27]. This demonstrates the validity of this approach for identifying genes undergoing selection.

Fig 1E shows the histogram of iHS values across the *CHRNA5-A3-B4* locus on chromosome 15. The summary statistics are given in Table 1. None of the three populations showed a proportion of extreme iHS values that was significantly different than predicted by permutation. In addition, none of the SNPs with extreme values included any of the SNPs previously found to be associated with nicotine dependence (Table 2). The presence of a significant enrichment of extreme Tajima’s D values in this region, however, suggests that if positive selection in this region occurred, it may have occurred long enough ago such that the long haplotypes required for iHS have broken down by recombination.

Fig 1F shows a histogram of the iHS values for the *CHRNB3-A6* cluster on chromosome 8. Summary statistics are shown in Table 1. Both the AFR and EUR populations show an excess of extreme iHS values (26.1% and 23.6%, respectively). By contrast, in the ASN population, there were few windows of extreme iHS scores and the overall proportion was not significantly different from the negative control. Several SNPs with extreme iHS values in these populations are contained within bins previously shown to be associated with either nicotine or cocaine dependence phenotypes [17,18]. Table 3 lists the SNPs from the LD bin showing genome-wide significance for reduced risk for nicotine dependence and provides the iHS value for rs1451240, the tag SNP. While there are several extreme values in AFR in the middle of this gene cluster, these values do not overlap with known SNPs related to nicotine dependence in this region. In EUR, 13 SNPs in this LD bin, including the tag SNP rs1451240, have extreme iHS values. All have positive values, indicating the presence of unusually long haplotypes containing the ancestral allele suggesting that the ancestral allele, which is associated with a greater risk of nicotine dependence is being favored by selection. An LD bin in the *CHRNB3-A6* region bin that has been shown to have SNPs significantly associated with increased risk for cocaine dependence [17] also contained an abundance of SNPs with extreme iHS values (S1 Table). This bin is fairly large and spans the entire *CHRNB3-A6* cluster. It contains rs4952 and rs4953, two low frequency synonymous variants in *CHRNB3* that have previously been reported to be associated with lower risk for nicotine dependence [10]. All SNPs in the bin are present at around 10% in AFR and 4% in EUR but absent in ASN, possibly explaining the lack of extreme iHS values in the ASN population. Overall, 40% of the SNPs in this bin showed extreme values in AFR and 15% of the SNPs in this bin showed extreme values in EUR. As these SNPs are absent from the ASN population, none showed extreme iHS values. The dense clustering of extreme iHS values in AFR and EUR is rarely expected under a neutral model, and is consistent with the hypothesis of the action of recent selection. In EUR, all of the SNPs in this LD bin with extreme iHS values had positive values and all were shared with AFR. Thus, in both populations, the ancestral allele associated with increased risk for nicotine dependence and decreased risk for cocaine addiction is being favored.

### Nicotine addiction and cognitive function

The Tajima’s D analysis and integrated haplotype score both indicate that the *CHRNB3-A6* cluster is undergoing selection and in particular, the iHS scores suggest that it is the risk allele for nicotine dependence on chromosome 8 that is under positive selection. As it seems unlikely that risk for nicotine dependence is the phenotype undergoing selection, and because nicotinic receptors are involved in memory and learning, we hypothesized that a phenotype related to memory or learning, such as attention, might be the phenotype being selected.

To test this possibility, we obtained genotype and cognitive phenotype data from the Collaborative Study of the Genetics of Alcoholism (COGA). Using this dataset, we tested the association between genotypes and three of the most relevant phenotypes, namely scores on the Wechsler Adult Intelligence Scale (WAIS) Block Design, WAIS Digit Symbol and WAIS Information tests. These tests were designed to measure aspects of perceptual organization, processing speed and verbal comprehension, respectively [29].

Table 4 summarizes our findings for the top SNPs associated with WAIS Digit Symbol test. No other neurocognitive phenotypes besides WAIS digit symbol had SNPs with significant values in the *CHRNB3-A6* region and none of the three neurocognitive phenotypes had a significant association with SNPs in the region of *CHRNA5-A3-B4* on chromosome 15 (not shown). Of the 17 SNPs in the *CHRNB3-A6* region on chromosome 8, one SNP–rs7017612—passed multiple test correction (p≤0.003) for association with the score on the WAIS Digit Symbol test (β = 0.43, p = 0.003). rs7017612 lies in the intergenic region between *CHRNB3* and *CHRNA6*. This SNP is highly correlated with rs6474413 (r^2^ = 0.75; D’ = 0.95), a SNP tagging the genome-wide significant bin for decreased risk for nicotine dependence. These data suggest a modest association between genotype at these SNPs one measure of cognitive function.

**Table 4:** Association results with SNPs in *CHRNB3-A6* region and Scores on the WAIS Digit Symbol test.

SNP	β	SE	p-value
**rs7017612**	**0.43**	**0.19**	***0*.*003***
**rs6982753**	**0.39**	**0.20**	***0*.*009***
**rs10958725**	**0.25**	**0.18**	***0*.*035***
**rs13273442**	**0.28**	**0.18**	***0*.*036***
**rs4950**	**0.24**	**0.18**	***0*.*038***
**rs1530848**	**0.24**	**0.18**	***0*.*038***
**rs6474413**	**0.25**	**0.18**	***0*.*038***
rs10107450	0.10	0.19	0.064
rs16891620	0.27	0.25	0.066
rs2196128	0.22	0.20	0.066
rs1530847	0.23	0.20	0.104
rs16891530	0.19	0.42	0.414
rs4952	0.19	0.42	0.414
rs7815274	0.19	0.42	0.426
rs10109429	0.07	0.31	0.494
rs13270610	-0.16	0.35	0.632
rs16891604	0.23	0.42	0.826

Beta, Standard Error (SE), and P-value for all SNPs in the *CHRNB3-A6* region of the COGA family GWAS and their association with scores on the WAIS Digit Symbol test. Covariates used were age, sex and FTND score. For all tests, 492 individuals were used for the association test.

A second SNP in the *CHRNB3-A6* region–rs6982753- had a nominal p-value with the WAIS Digit Symbol phenotype before multiple test correction and almost passed the multiple test correction (p = 0.009). Interestingly, this SNP has an r^2^ of 0.91 with rs892413 (β = 0.39, p = 0.008), a SNP that has previously been associated with increased risk for cocaine dependence [17].

## Discussion

Many studies have demonstrated that risk for nicotine addiction has a genetic component. We performed two tests of selection on chromosomal regions containing the genes encoding five nicotinic receptor subunits and each of these analyses indicate that selection likely occurred at the *CHRNB3-A6* locus. Both the Tajima’s D test and iHS point to an ongoing sweep in humans on chromosome 8. In the case of the *CHRNB3-A6* locus, all of the extreme values in the Tajima’s D analysis were positive. High positive Tajima’s D values occur when there is an excess of variants in a region with intermediate allele frequencies. This can occur in either balancing selection or ongoing positive selection. We also found extreme iHS values in the *CHRNB3-A6* locus. This region fulfills the criteria for a sweep laid out by Voight and colleagues [30], i.e. clustering of extreme iHS values. Extreme values of iHS are unlikely under simple demographic models, and thus can indicate the action of an ongoing selective sweep. Despite the fact that none of the populations had significantly different p-values from the negative control in the chromosome 8 region, a few key SNPs did have extreme values. Together, these data imply that the Tajima’s D analysis is picking up on ongoing positive selection rather than balancing selection.

Several SNPs in the *CHRNB3-A6* locus on chromosome 8 have previously been associated with a decreased risk of nicotine dependence [18]. One of these, rs1451240, was present in a window that showed extreme values in both the Tajima’s D test and iHS. The extreme positive iHS value in the window including rs1451240 indicates that the haplotype containing the ancestral allele is being positively selected. As the derived allele provides protection from nicotine addiction, this suggests that it is the allele that is associated with a greater risk of nicotine dependence that is being selected. Since highly concentrated sources of nicotine were not present in the ancestral environment, it seems likely that this phenotype of nicotine dependence would have hitchhiked along with a more beneficial phenotype. One challenge with this region is that it is approximately 1,500,000 bp away from the centromere of chromosome 8. This could be affecting the results by some unknown mechanism. However, the region including the nicotinic receptors on chromosome 8 was among the top 5% of iHS scores among all regions tested in the genome.

Selective pressures in our ancestral environments were likely not on addiction, but rather on behaviors that were biologically rewarding (i.e. mate or food finding, avoidance of harmful stimuli). Given the role of nicotine in neurological function, it is possible that, in the case of nicotine addiction, the phenotype on which natural selection was working was related to enhancements in memory or cognition. The addiction phenotype would have hitchhiked along because it acts through the same or related mechanisms. The addiction phenotype was likely not selected against in ancestral environments because the availability and opportunity for prolonged use of purified drugs was negligible.

To test this possibility, we assessed the association of SNPs in the *CHRNB3-A6* locus with scores on WAIS tests of memory and cognitive function. Our analysis of the individuals in the COGA dataset suggests that one SNP, rs7017612, which lies in the intergenic region between *CHRNB3* and *CHRNA6*, is associated with increased score on the WAIS Digit Symbol test. This test is thought to largely measure processing speed, but also, to some extent, memory. rs7017612 itself has not been reported to be associated with nicotine dependence. However, it is in moderately high LD (r^2^ = 0.75) with rs6474413, a SNP tagging the genome-wide significant bin for decreased risk for nicotine dependence. Thus, our data are consistent with the possibility that improved performance on this particular cognitive test is modestly associated with a *decreased* risk for nicotine dependence and that alleles of SNPs in these regions have effects on a subset of cognitive pathways best captured here by the WAIS digit symbol test. It is possible, however, that a function other than addiction or cognition is the true phenotype undergoing natural selection at these loci.

Genetic studies of nicotine addiction have identified an inverse relationship between the risk for nicotine addiction and the risk for cocaine addiction. For instance, the minor allele of rs16969968, a missense variant in *CHRNA5*, increases risk for the development of nicotine dependence, and independently decreases risk for cocaine dependence [15]. One hypothesis would be that the true underlying selective pressure is on cocaine related phenotypes or a characteristic that affects cocaine related reward pathways in the brain and that the alleles’ effect on nicotine dependence is merely an accidental consequence. However, caution must be used when interpreting this information, given that all drugs of addiction similarly affect the dopaminergic reward pathways.

Another alternative hypothesis is that the selective pressure at this locus was on social behavior. Cocaine addiction is characterized by a dampened reward response to social interaction, meaning that it inhibits the positive emotions that accompany social interaction or feelings of belonging. A recent study demonstrated that cocaine users process social gaze (joint attention on an object) differently than controls, resulting in a reduced activation of the reward system during social interactions [31]. Using fMRI, these authors showed that cocaine users had decreased activation of the medial orbitofrontal cortex, a region of the brain central for reward processing. If alleles that alter cocaine dependence risk alter an individuals’ natural reward system during social interactions, these observations could explain why alleles that protect against cocaine dependence could have provided advantage to carriers in the ancestral environment. Since nicotine sensitizes the animal to the effects of cocaine, which blunts the reward of social interactions, alleles that reduced the ability of nicotine to enhance the effects of cocaine would have undergone positive selection. In this scenario, the nicotine and cocaine dependence phenotypes are not hitchhiking with memory or learning, but rather with phenotypes protecting against antisocial and therefore maladaptive behavior.

There was also moderate evidence for selection at the *CHRNA5-A3-B4* locus. In particular, rs16969968, the SNP that encodes the missense mutation in α5, that is strongly associated with risk of nicotine dependence, lies in a sliding window exhibiting a high Tajima’s D score. The iHS analysis of this locus did not provide evidence for selection. This could indicate that the selective pressure exerted on this locus is older that that seen for the *CHRNB3-A6* locus and as such has allowed extended haplotypes to be broken down by recombination. In this scenario, Tajima’s D would still be extreme while iHS scores in the region might be less so. This may be particularly true if the selective pressure being exerted on the *CHRNB3-A6* locus is ongoing.

Here we have used two statistical tests of selection and uncovered evidence of positive selection at the nicotinic receptors on chromosome 8 chromosome 15. Multiple drug-related phenotypes are associated with SNPs in or near these loci, however for several reasons it is unlikely that these phenotypes are the direct targets of this selective pressure. We have proposed two possible explanations 1) phenotypes related to memory and learning and 2) phenotypes related to social behavior. We were only able to discover a modest association with memory-related phenotypes, likely due to the small sample size. We also are as of yet unable to test this second hypothesis because we do not have data in our sample for a phenotype measuring sociality. However this work is the first to explicitly describe signs of natural selection acting on loci underlying substance dependence phenotypes.

## Materials and Methods

To determine whether the nicotinic receptor loci are under selection, we used Tajima’s D, and integrated haplotype score (iHS) to examine the landscape of natural selection at three loci previously demonstrated to harbor genetic variants contributing to the risk of nicotine dependence. These tests have different but complementary strengths. Tajima’s D test functions best on recently completed selective sweeps. There are many variables that contribute to how far in the past a sweep can be detected, such as how extreme the sweep was in the first place. Both the mutation rate and the recombination rate affect it as well and vary widely across the genome making generalizations difficult. By contrast, integrated haplotype score iHS functions best for detecting sweeps in progress with alleles at intermediate frequencies, mainly in the range of or after the separation of European, Asian and African populations, during the agricultural phase of human evolution.

We utilized 1000 Genomes data for these analyses. Data was obtained from the 1000 genomes website (http://www.1000genomes.org/data), a third party source for population whole genome sequence data. The populations were grouped into EUR (GBR, TSI, CEU, FIN), ASN (CHS, CHB, JPT), and AFR (YRI, LWK, ASW). All methods were calculated for the same regions: the *CHRNA5-A3-B4* region on chromosome 15q25, the *CHRNB3-A6* region on chromosome 8p11, the *LCT* region as a positive control on chromosome 2q21, and ten intergenic negative control regions where applicable.

### Tajima’s D test

Tajima's D is a method of addressing the frequencies of variant sites, based on the expectation that under neutrality, different estimates of expected diversity (θ) should be equal. Tajima’s D tests for a skew in the frequency spectrum by comparing two estimates of θ – the number of segregating sites (S), and pairwise nucleotide diversity (π) [32]. Extreme positive values can indicate either balancing selection or population subdivision, and extreme negative values can indicate positive selection or population growth [33]. If the same skew is detected across the genome, the effect is likely due to demography, whereas if the skew is localized to a few loci, selection is more likely to be occurring.

In a review, Garrigan & Hammer [34] have combined published data for Tajima’s D values from 65 autosomal loci. They find the mean value for Africans is slightly negative (-0.20) and for non-Africans is slightly positive (0.13). Overall, the values range from approximately -2 to 2. As such, we have taken Tajima’s D values above 2 or below -2 to count as extreme values, as this represents the 95% confidence interval of values in our data.

Tajima’s D was calculated using the program Variscan [35]. After an exploratory data analysis of window size, we used a sliding window size of 1000 bp, and window increments of 100 bp for the analysis. Smaller window sizes resulted in too few SNPs in a window to calculate Tajima’s D, while larger windows made it much harder to narrow down specific SNPs that may be the ultimate target of selection. Variscan outputs a file giving the Tajima’s D value for every window of the specified bp size on the sliding scale [35]. These values were then superimposed onto graphs of the regions.

### Integrated Haplotype Score

iHS is a measure of whether a SNP is on an unusually long haplotype carrying the ancestral or derived allele. It compares the rate of haplotype decay between haplotypes carrying either the ancestral or derived allele at a given site, referred to as the core SNP. Haplotypes whose core SNP is under selection will be unusually long compared to those evolving neutrally. Long haplotypes with derived alleles are indicated by negative iHS values and those with ancestral alleles are indicated by positive iHS values. Under neutrality, extreme scores are distributed throughout the genome, however under selection, they are clustered across the selected region [30]. iHS is a good method for detecting directional selection, especially in a sweep that is in its early phases. We used the program WHAMM to calculate this statistic [30].

The haplotype decay is calculated until the extended haplotype homozygosity (EHH) reaches 0.05. EHH is defined as “the probability that two randomly chosen chromosomes carrying the core haplotype of interest are identical by descent for the entire interval from the core region to point x” [36] (p. 833). Long haplotypes with derived alleles are indicated by negative iHS values and those with ancestral alleles are indicated by positive iHS values. Under neutrality, extreme scores are distributed throughout the genome, however under selection, they are clustered across the selected region [30].

First, we extracted the desired regions from the 1000 Genomes dataset. We then selected known SNPs within each region, and extracted a region of plus or minus 2000 SNPs around that SNP, except in the case of *CHRNB3-A6* where we selected plus or minus 2500 SNPs. We constructed recombination maps using cM maps provided by the SHAPEIT2 program [37]. Ancestral alleles were determined using the latest version of Seattleseq (http://snp.gs.washington.edu/SeattleSeqAnnotation137/). Phased haplotypes were coded as number of copies of the derived allele. All positions in which the derived allele could not be determined unambiguously (i.e. C/G or A/T SNPs) as well as those without known chimp alleles were removed from further analyses. All analyses were run on each population separately. As iHS is greatly influenced by SNP allele frequency, iHS values from WHAMM were standardized using the average and standard deviation of all SNPs on chromosome 15 and 8 binned by allele frequency such that the average iHS value for each bin after standardization was identical. We excluded SNPs with a minor allele frequency less than 5% because low frequency SNPs are difficult to normalize accurately. After removing these SNPs, extracting just the desired gene regions, and removing those with MAF of <0.05, there were ~150–350 SNPs per region, depending on the population. Standardization was done separately for each population using population specific averages and standard deviations. iHS values were then superimposed onto graphs of the regions.

The haplotype on which a beneficial allele resides tends to be significantly longer than the other haplotypes at the same frequency in the population when adjusted for the recombination background. However, long haplotypes tend to occur in regions with low recombination, and these can be confused with genuine genomic signals of positive selection [38]. This is why WHAMM attempts to control for recombination by requiring the input of a cM map. The map we used here was the cM map for imputation available on the website for the program SHAPEIT2.

Candidate regions of positive selection were defined as genomic regions containing an uncharacteristic clustering of SNPs with high iHS statistics. This was quantified as the proportion of SNPs with |iHS| > 2 in the four regions of interest. Candidate regions of positive selection were identified as containing any SNP with an iHS score of |iHS| > 2, as this corresponds to the top ~5% of all scores. The iHS value at a SNP “measures the strength of evidence for selection acting at or near that SNP” however does not provide a formal significance test (Voight et al. 2006).

### Association Analyses

COGA recruited subjects in Hartford, Connecticut; Indianapolis, Indiana; Iowa City, Iowa; New York City, New York; San Diego, California; St Louis, Missouri; and Washington, DC. For inclusion in SAGE, cases had to meet lifetime criteria for DSM-IV alcohol dependence, the majority of cases were recruited from alcoholism treatment centers. Control subjects, were both biologically unrelated to cases, and had consumed alcohol but never experienced any significant alcohol or drug-related problems, according to the Semi-Structured Assessment for the Genetics of Alcoholism (SSAGA) [18]. The COGA sample utilized in this study consisted of family GWAS data from 2102 European-Americans [14]. De-identified data from the Collaborative Studies on the Genetics of Alcoholism (COGA) were used. All participants in COGA provided written informed consent for genetic studies and agreed to share their DNA and phenotypic information for research purposes. The Washington University Human Research Protection Office granted approval for data to be used for this study.

COGA administered a variety of neuropsychological tests to its subjects including the three used here: Wechsler Adult Intelligence Scale-Revised (WAIS-R) Block Design, WAIS Digit Symbol, and WAIS Information. In total there were 1247 European-Americans with these neuropsychological phenotypes. However, the overlap between this number and those with family GWAS data was 492. Therefore, our analyses were performed on 492 subjects. In WAIS Block Design, the subject replicates models or pictures of two-color designs with blocks. In WAIS Information, the subject answers a series of questions about factual information. In WAIS Digit Symbol, the subject writes down as quickly as possible the symbols that correspond to a series of numbers.

SNPs in the region of the nicotinic receptor clusters on chromosomes 8 and 15 were tested for association with the scaled scores of neuropsychological phenotypes in European-Americans from the COGA study using an additive linear mixed effects (LME) model with the lmepack.batch function as implemented in the GWAF package in R using age, sex and FTND score as covariates [39]. Neither alcohol nor cocaine symptom count were significant covariates in the analysis and were thus not included in the analysis. The GWAF package enables association testing with the ability to include individuals from families by correcting for relatedness specified in a pedigree file.

## Supporting Information

S1 Table Tajima’s D and iHS values for SNPs correlated with rs9298626 within the *CHRNB3-A6* region. All SNPs were highly correlated (r^2^>0.9) with the SNP rs9298626.
(PDF)
