# Supplementary material for: Positive Selection on Loci Associated with Drug and Alcohol Dependence
Source: PLoS One. 2015 Aug 13;10(8):e0134393. doi: 10.1371/journal.pone.0134393 (PMC4536217; doi:10.1371/journal.pone.0134393)
Supplement: S1 Table — All SNPs were highly correlated (r2>0.9) with the SNP rs9298626. (PDF) [file pone.0134393.s001.pdf]

SNP	dbSNP Functional Annotation	Frequency In 1000 Genomes EUR	iHS Value EUR	Frequency In 1000 Genomes ASN	iHS Value ASN	Frequency In 1000 Genomes AFR	iHS Value AFR
rs62516708	intergenic	0.0396	0.299	0	n/a	0.0955	0.867
rs13439675	intergenic	0.0383	-0.113	0	n/a	0.0163	0.268
rs9298625	intergenic	0.0396	0.019	0	n/a	0.0955	1.217
**rs9298626**	intergenic	0.9604	0.518	0	n/a	0.9045	-0.447
rs1451241	intergenic	0.0383	0.565	0	n/a	0.0163	0.362
rs1451242	intergenic	0.0396	0.671	0	n/a	0.0955	1.329
rs28616109	intergenic	0.0396	0.729	0	n/a	0.0955	1.317
rs62516714	intergenic	0.9591	-0.233	0	n/a	0.9248	-0.717
rs76304348	intergenic	0.0383	0.803	0	n/a	0.0041	-0.493
rs62516738	intergenic	0.0383	0.976	0	n/a	0.0041	-0.077
rs62516743	intronic	0.0396	1.388	0	n/a	0.0061	0.997
rs62516744	intronic	0.0383	1.292	0	n/a	0.0447	1.339
rs62516747	intronic	0.9617	-0.688	0.9615	0.133	0.8943	**-2.155**
rs62516749	intronic	0.9617	-0.686	0	n/a	0.9024	-1.626
rs80157946	intronic	0.0383	1.362	0	n/a	0.0996	**2.651**
rs74675009	intronic	0.9617	-0.701	0	n/a	0.9004	-1.696
rs62516750	intronic	0.9617	-0.893	0	n/a	0.9004	-1.822
rs62516752	intronic	0.0383	1.573	0	n/a	0.0996	**2.691**
rs62516754	intronic	0.9617	-0.893	0	n/a	0.9004	-1.731
rs62516757	intronic	0.9617	-0.996	0	n/a	0.9004	-1.741
rs62516758	intronic	0.9617	-0.996	0	n/a	0.9004	-1.741
rs62516759	intronic	0.9617	-0.996	0	n/a	0.9004	-1.741
rs62518160	intronic	0.9617	-0.996	0	n/a	0.9004	-1.741
rs62518180	intronic	0.9617	-1.016	0	n/a	0.9004	-1.75
rs62518181	intronic	0.9617	-1.016	0	n/a	0.9004	-1.75
rs62518182	intronic	0.9617	-1.016	0	n/a	0.9004	-1.75

rs62518183	intronic	0.9617	-1.016	0	n/a	0.9146	-1.788
rs62518184	intronic	0.9644	-1.044	0	n/a	0.9085	-1.759
rs76121545	intronic	0.0383	**2.729**	0	n/a	0.0061	**2.138**
rs79103618	intronic	0.0383	**2.729**	0	n/a	0.1016	**2.934**
rs77200520	intronic	0.9617	-1.943	0	n/a	0.9085	-1.719
rs74931311	intronic	0.9617	-1.943	0	n/a	0.9085	-1.719
rs62518192	intronic	0.9617	-1.943	0	n/a	0.9004	-1.774
rs62518193	intronic	0.0383	**2.729**	0	n/a	0.0061	**2.17**
rs62518194	intronic	0.0383	**2.729**	0	n/a	0.0996	**2.698**
rs62518196	intronic	0.0383	2.733	0	n/a	0.0061	2.093
rs62518201	intronic	0.9617	-1.658	0	n/a	0.9045	**-2.623**
rs62518202	intronic	0.9617	-1.657	0	n/a	0.9004	**-2.705**
rs62518203	intronic	0.0383	**2.414**	0	n/a	0.0996	**3.798**
rs62518204	intronic	0.9617	-1.657	0	n/a	0.9004	**-2.705**
rs7008048	intronic	0.0383	**2.414**	0	n/a	0.0996	**3.461**
rs62518206	intronic	0.9617	-1.657	0	n/a	0.9004	**-2.449**
rs62518210	intronic	0.0383	**2.398**	0	n/a	0.0996	**3.529**
rs62518211	intronic	0.0383	**2.398**	0	n/a	0.0996	**3.529**
rs62518212	intronic	0.0383	**2.398**	0	n/a	0.0996	**3.529**
rs62518213	intronic	0.0383	1.77	0.0035	-0.416	0.0061	1.096
rs77930111	intronic	0.9617	-1.058	0	n/a	0.9004	**-2.119**
rs59569717	intronic	0.9617	-1.058	0	n/a	0.8963	**-2.244**
rs58205452	intronic	0.0383	1.755	0	n/a	0.0996	**3.124**
rs7832104	intronic	0.0383	1.721	0	n/a	0.0996	**3.338**
rs118073227	intronic	0.0383	1.722	0	n/a	0.0061	1.093
rs57499403	intronic	0.9617	-1.002	0	n/a	0.9024	-1.979
rs6997994	intronic	0.9617	-1.002	0	n/a	0.9004	-1.911
rs7002613	intronic	0.9617	-1.002	0	n/a	0.9004	**-2.044**
rs7002907	intronic	0.9644	-0.822	0	n/a	0.9045	-1.839
rs77259784	intronic	0.0356	1.495	0	n/a	0.0996	**2.927**

rs55817013	intronic	0.9617	-0.754	0	n/a	0.9004	-1.573
rs4952	intronic	0.0383	1.393	0	n/a	0.0061	0.953
